# Smad3 Signatures in Renal Inflammation and Fibrosis

**DOI:** 10.7150/ijbs.71595

**Published:** 2022-03-28

**Authors:** Wenjing Wu, Xiaoqin Wang, Xueqing Yu, Hui-Yao Lan

**Affiliations:** 1 Guangdong-Hong Kong Joint Laboratory for Immunological and Genetic Kidney Disease, Department of Pathology, and Guangdong Cardiovascular Institute, Guangdong Academy of Medical Sciences, Guangdong Provincial People's Hospital, Guangzhou, China.; 2 Departments of Medicine & Therapeutics, Li Ka Shing Institute of Health Sciences, and Lui Che Woo Institute of Innovative Medicine, The Chinese University of Hong Kong, Hong Kong, China.; 3 Hubei University of Chinese Medicine, Wuhan, China.; 4 Department of Nephrology, Hubei Provincial Hospital of Traditional Chinese Medicine, Hubei Province Academy of Traditional Chinese Medicine, Wuhan, China.; 5 The Chinese University of Hong Kong-Guangdong Academy of Sciences/ Guangdong Provincial People's Hospital Joint Research Laboratory on Immunological and Genetic Kidney Diseases, The Chinese University of Hong Kong, Hong Kong, China.

**Keywords:** Smad3, renal inflammation and fibrosis, miRNAs, lncRNAs

## Abstract

Renal inflammation and fibrosis are key pathological features of acute kidney injury (AKI) and chronic kidney disease (CKD). Smad3 is a critical mediator of TGF-β signaling and plays a pathogenic role in both renal inflammation and fibrosis. Smad3 can be activated not only by TGF-β1 but also by many stress molecules including angiotensin II (Ang II), advanced end products (AGEs), and C-reactive protein (CRP) under disease conditions. In addition, Smad3 can interact with other signaling pathways, such as the ERK/p38 MAPK and NF-κB pathways, to mediate renal inflammation and fibrosis. Mechanistically, Smad3 transcriptionally regulates many downstream target genes including microRNAs and long non-coding RNAs to cause cell death, inflammation, and fibrosis. Thus, targeting Smad3 or its downstream genes specifically related to renal inflammation and fibrosis should provide a novel therapeutic strategy to combat kidney diseases.

## Introduction

Kidney disease has become a major public health problem worldwide. Chronic kidney disease (CKD) affects more than 10% of the population worldwide 1. Renal inflammation is a common manifestation of acute kidney injury (AKI) and CKD 2, 3 and may be a driving force from AKI to CKD progression. Fibrosis is a common pathway of progressive CKD that finally leads to the end-stage renal disease. Thus, renal fibrosis accompanied by active inflammation is a major pathological feature in progressive kidney disease 3. Like Chinese Yin and Yang, transforming growth factor-β1 (TGF-β1) signaling plays a diverse role in renal inflammation and fibrosis 4-7. It is well documented that TGF-β is a potent anti-inflammatory cytokine and immune regulator that play a protective role in renal inflammation. In the other hand, TGF-β also exerts its pathogenic role in renal fibrosis 4-7. Upon TGF-β binds to its receptors, it triggers activation of downstream signaling pathways including Smad and non-Smad-dependent pathways. Of them, the canonical Smad pathway is a key regulatory pathway in the pathogenesis of renal inflammation and fibrosis. Of the Smad signaling molecules, Smad3, together with Smad2, is the major receptor-associated Smads. Smad3 has a highly conserved region at the N-terminal and C-terminal, termed mad-homology domain1 (MH1) region and MH2 region. MH1 is mainly associated with DNA binding, while the MH2 region has phosphorylation sites activated by TGF-β1 and has specific sequences that determine their binding to TGF-β1 signal receptors 8, 9.

Smad3 is confirmed to be a major downstream signaling molecule of TGF-β1 in mediating organ inflammation and fibrosis 4. Smad3 can be activated by many other stress molecules including angiotensin II (Ang II), advanced end products (AGE), and C-reactive protein (CRP). In this review, we focus on the molecular mechanisms of Smad3 in regulating renal inflammation and fibrosis. We also describe the downstream Smad3 signature genes including Smad3-dependent microRNAs and long non-coding RNAs, which regulate renal inflammation and fibrosis. In addition, the new therapeutic approaches for kidney disease by targeting Smad3 signaling, as well as Smad3-dependent microRNAs and lncRNAs are also described.

## Regulatory role and mechanisms of Smad3 in renal fibrosis

**Figure 1:**
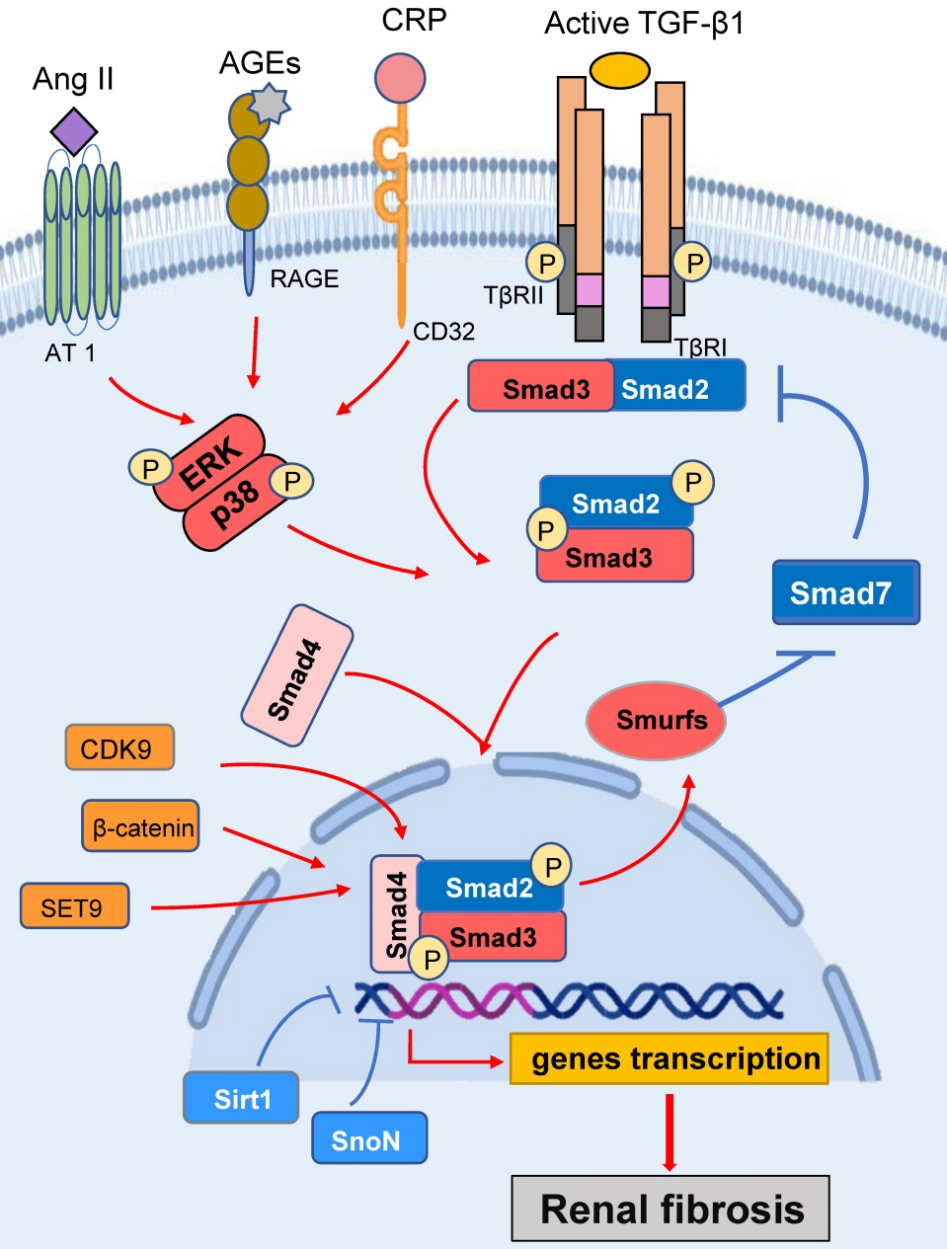
** Smad3 signaling and crosstalk pathways in renal fibrosis.** After binding to TβRII, TGF-β1 activates the TβRI-kinase which phosphorylates Smad3. The phosphorylated Smad3 translocates into the nucleus and regulates the target gene transcription. Smad7 is an inhibitory Smad that functions to block Smad3 activation by degrading the TβRI and preventing phosphorylation of Smad3.Ang II, AGEs and CRP can activate TGF-β1-independent signaling via the ERK/p38/ MAPK crosstalk pathway. Red arrows/ symbols represent pathogenic or positive regulation pathway, while blue lines/ symbols indicate protective or negative regulation pathways in fibrosis.

TGF-β1 has long been known as a key mediator in the pathogenesis of renal fibrosis by activating the downstream Smad proteins, especially Smad3. Once Smad3 becomes activated in response to TGF-β1 and other stress molecules such as Ang II, AGEs, and CRP, it can translocate to the nucleus to directly bind to DNA sequences and regulate the target genes (Figure 1). It is known that many fibrogenic genes responsible for the fibrogenesis including collagen synthesis and epithelial-mesenchymal transition (EMT) are Smad3-dependent 10, 11. Thus, Smad3 plays a critical role in the development of renal fibrosis in many kidney diseases. An essential role for Smad3 in fibrogenesis is confirmed by the findings that deletion of Smad3 from mice can suppress renal fibrosis in a number of rodent models, including diabetic nephropathy 12, obstructive kidney diseases 13, hypertensive nephropathy 14, and drug-associated nephropathy 15.

Smad3 can also regulate Smad7 to play a role in renal fibrosis. It is well established that Smad7 is an inhibitory Smad that is induced by Smad3 transcriptionally but exerts its negative feedback mechanism to maintain the homeostasis of TGF-β/Smad signaling 5, 16-19. In normal situations, renal Smad7 is abundant and exerts its negative feedback mechanism by causing degradation of Type I TGF-β receptor (TβRI) via an ubiquitin proteasome degradation mechanism, thereby preventing the recruitment and phosphorylation of Smad3 4. Under disease conditions, Smad3 is overreactive and can also induce a number of E3 ubiquitin ligases such as the Smad ubiquitination regulatory factor 1 (Smurf1), Smad ubiquitination regulatory factor2 (Smurf2), and arkadia, which physically interact with Smad7 and cause an ubiquitin-dependent degradation of renal Smad7 protein 20, 21, resulting in enhanced TGF-β/Smad3 signaling and progressive renal fibrosis 20. This is further supported by the findings that mice lacking Smad7 largely promote activation of Smad3 signaling and progressive renal fibrosis in both obstructive nephropathy and diabetic kidney disease 22, 23.

Renal fibrosis is characterized by a loss of renal tubules and the accumulation of extracellular matrix (ECM). Myofibroblasts are an active form of fibroblasts that are generally considered to be the main source of ECM production during renal fibrosis 24, 25. Many studies show that Smad3 has an important role in the transformation of bone marrow-derived fibroblasts in the kidney as genetic disruption of Smad3 inhibits the activation of bone marrow-derived fibroblasts in the kidney in response to obstructive kidney injury *in vivo* and suppresses monocyte-to-fibroblast transition *in vitro* 26. Excitingly, our recent studies have also demonstrated that macrophage-myofibroblast transition (MMT) is a major source of myofibroblast origin (> 60%) that occurs locally within the fibrosing kidney and is regulated by Smad3 27-29. Smad3 is required for the efficient transition of recruited macrophages to become collagen I-producing α-SMA^+^ myofibroblasts within the injured kidney. In addition, the protection from obstructive kidney fibrosis seen in Smad3^-/-^ chimeric mice provides further evidence that bone marrow-derived macrophages make a substantial contribution to the development of renal fibrosis via the Smad3-dependent MMT process 28, 30. Indeed, bone marrow-derived M2-type pro-fibrotic macrophages are highly proliferative and contribute to renal fibrosis in the UUO kidney 31. It is proposed that bone marrow-derived M2 macrophages can enter the injured kidney and then transdifferentiate into collagen-producing α-SMA^+^ myofibroblasts which is under tight control of TGF-β/Smad3 signaling 32. By using single cell RNA sequence analysis, we reveal that TGF-β1 induces MMT in bone marrow-derive macrophages via the Smad3-Src-POU4F1 pathway as Smad3 can bind Src and POU4F1 promoters to induce MMT and targeting this pathway can block MMT and renal fibrosis *in vitro* and *in vivo* 30, 33.

Ang II and AGEs are also able to induce renal fibrosis by activating Smad3 signaling via TGF-β-dependent and-independent pathways (Figure 1). Ang II is able to induce TGF-β1 expression and then activate TGF-β1/Smad3 signaling, which can lead to fibrosis. In addition, a significant finding shows that Ang II can also directly activate Smad3 to induce expression of connective tissue growth factor (CTGF) and collagen I through the AT1-ERK/p38 MAPK crosstalk pathway 34. This is supported by the findings that addition of Ang II is able to induce Smad3 phosphorylation in tubular epithelial cells (TECs) lacking the TGF-β1 gene, and that blockade of the AT1 receptor, ERK1/2, and p38, is capable of inhibiting Ang II-induced activation of Smad3 and CTGF 34. This notion is further supported by evidence of knockdown Smad3 to block Ang II-mediated EMT 34. Ang II-induced overactivation of TGF-β1/Smad3 signaling is also associated with the loss of renal Smad7, which is mediated by a Smurf2-dependent ubiquitin degradation mechanism 35.

It is now well accepted that AGEs are key mediators in diabetic nephropathy. Accumulation of AGEs closely correlates to CTGF expression and EMT. Indeed, the Smad-binding elements are found in the CTGF promoters 36, suggesting that Smad3 may mediate fibrosis by inducing CTGF expression. Because AGEs are able to activate Smad3 via TGF-β1-dependent and independent mechanisms 4, 5 (Figure 1), it is generally believable that AGE-induced CTGF expression via the TGF-β1-independent Smad3 signaling pathway as addition of AGEs is able to stimulate a rapid phosphorylation of Smad2/3, ERK1/2, and p38 and CTGF expression in TECs lacking TGF-β1 gene 37, 38.

C-reactive protein (CRP) acts as one of the most essential inflammatory marker and mediator in various chronic diseases 39, 40 (Figure 1). It mediates renal fibrosis by inducing the early (15 mins) and the late phase (24 hrs) of phosphorylation of Smad3 in HK-2 cells 41, 42. CRP can activate Smad3 to mediate renal fibrosis directly via the CD32b-ERK/p38 MAP kinase-crosstalk pathway and indirectly through the TGF-β1-dependent mechanism 41. This is also confirmed by deleting Smad3 to inhibit UUO-induced renal fibrosis in CRP transgenic mice 43.

Smad3 is also activated by other molecules (Figure 1). Of them, cyclin dependent kinases (CDKs), such as CDK9, can promote Smad3-regulating collagen I promoter activity 44. Ski-related novel protein (SnoN) is a nuclear protein that functions as a negative regulator of TGF-β1/Smad3 signaling 45. Activation of Smad3 can up-regulate Smurf2 and thus enhances the ubiquitin degradation of SnoN to exert the fibrogenic effects of TGF-β1/Smad3 signaling. Further study shows that Smad3 can repress SnoN transcription by binding to SIE sequences (triple CGACGG box) in the SnoN promoter 46, 47. β-catenin acts as a co-factor for Smad3 transcriptional activity. Targeted degradation of cytosolic β-catenin or inhibition of β-catenin binding to Smad3 blocks TGF-β1-induced EMT in murine renal tubular epithelial cells 48. Sirt1 belongs to a highly conserved family of NAD^+^‐dependent deacetylase and has been reported to deacetylate the lysine residues of a number of nuclear proteins. It has been reported that Sirt1 can bind to Smad3 to reduce the acetylation levels of Smad3 and inhibits renal fibrosis 49. It is also reported that the methyltransferase SET9 (also known as SETD7) can interact with the Smad3 N-terminal MH1 domain to increase Smad3 activity and upregulate α-SMA expression during renal fibrosis 50. CREB binding protein (CBP) is also a Smad3 coactivators, it can bind to the Smad complexes to transcriptionally regulate the downstream genes 51. CREB can competitively inhibit the binding of CBP to Smad3 52. Glycogen synthase kinase 3β (GSK3β) is a serine/threonine-protein kinase that inhibits the CREB activities while promoting CBP binding to Smad3 to facilitate renal fibrosis. In TGF-β1-treated renal TECs, the inhibition of GSK3β can enhance the activity of CBP recruitment to CREB and thus ameliorates renal fibrosis 52.

## Regulatory role and mechanisms of Smad3 in renal inflammation

**Figure 2:**
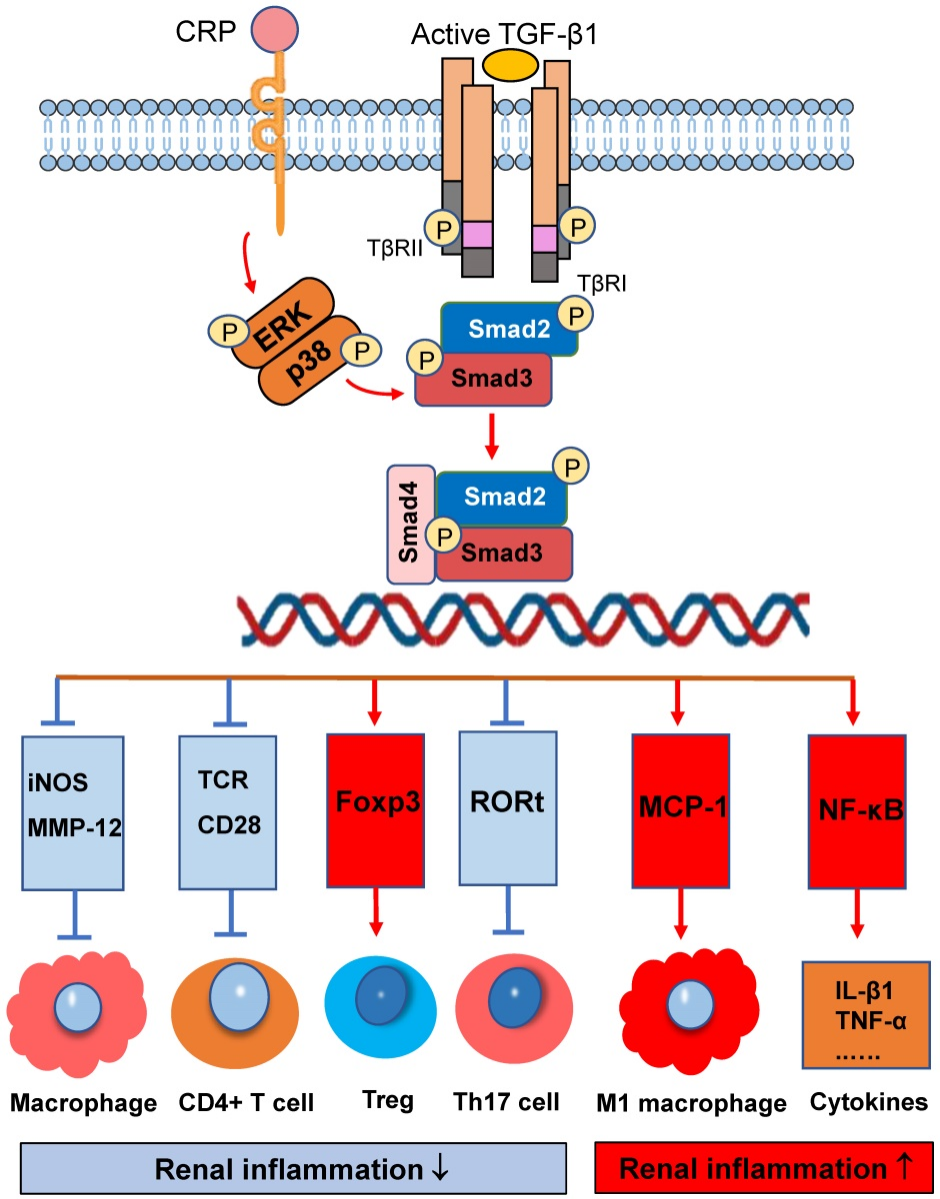
** Smad3 signaling and crosstalk pathways in renal inflammation.** Smad3 is a key regulator that diversely regulates renal inflammation by either inhibiting or promoting macrophage and T cell. Red arrows/symbols represent pathogenic or positive regulation pathway, while blue lines/symbols indicate protective or negative regulation pathways.

Infiltration of immune cells into tissues is a key pathogenetic event in many inflammatory diseases which can be diversely regulated by Smad3. As shown in Figure 2, activation of Smad3 plays a diverse role in immune cell activation and differentiation during renal inflammatory responses. Smad3 is a critical effector molecule of TGF-β1-mediated inhibition of macrophage activation as Smad3 is capable of inhibiting the promoter activities of iNOS and MMP-12 on macrophages 53. In addition, Smad3 also mediates the TGF-β-dependent inhibition of CD4 T-cell proliferation. Smad3 phosphorylation decreases T-cell receptor (TCR) activation, as well as impeding the effects of CD28 co-stimulation 54. It is now well defined that Smad3 is a downstream key regulator of TGF-β signaling in T cell immunity 55-57. It is well documented that Smad3 can bind and regulate expression of Foxp3 to promote Treg cell differentiation and functions in many immunologically-mediated kidney diseases including crescentic glomerulonephritis 58, 59. In addition, Smad3 is found to be part of a protein complex with RORt, leading to the inhibition of RORt transcriptional activity and then decline Th17 cell generation 60. Thus, activation of TGF-β/Smad3 signaling exerts its inhibitory effect of on immunologically-mediated diseases by promoting Treg while inhibiting Th17 responses. However, together with IL-6, activation of TGF-β/Smad3 plays an important role in the generation of Th17 cells 61. Thus, Smad3 is an important regulator in maintaining the balance between Treg and Th17 immune responses, which is supported by the findings that Smad3 deficiency resulted in defective Foxp3 induction but enhanced Th17 cell generation *in vitro* and *in vivo* 60.

On the other hand, activation of TGF-β/Smad3 signaling may also promote renal inflammation. There are two possible mechanisms responsible for this pro-inflammatory effect of TGF-β/Smad3 signaling. First, Smad3 may exert its chemotactic effect on the macrophage recruitment during renal inflammation as Smad3 can interact with macrophage chemotactic protein-1 (MCP-1) to promote macrophage-dependent renal inflammation 62. This is supported by the findings that mice lacking Smad3 remarkably suppress renal inflammation by reducing F4/80^+^ macrophages, together with CD4 and CD8 T cells in the diseased kidneys of obstructive nephropathy 26, 28, 30, 63, Ang II-induced hypertensive nephropathy 14, and ischemic-reperfusion AKI 64. Interestingly, recent studies also show that deletion of Smad3 from db/db and human CRP transgenic mice can inhibit renal inflammation by blocking MCP-1-dependent macrophage infiltration 65. Furthermore, deficiency of Smad3 shows to exert its inhibitory effect on NF-κB-driven renal inflammation as seen in many mouse models of kidney disorders 41, 43. It is likely that deletion of Smad3 may suppress expression of E3 ubiquitin-protein ligases such as Smurf1/Smurf2 that target Smad2, Smad7, and TβRI for degradation. Thus, protection of renal Smad7 from the E3-ligase-dependent ubiquitin degradation in Smad3 KO mice may result in upregulation of IκBα, an inhibitor of NF-κB signaling, thereby inhibiting NF-κB-driven renal inflammation 66, 67.

## Regulatory role and mechanisms of Smad3 in cell death during acute kidney injury

**Figure 3:**
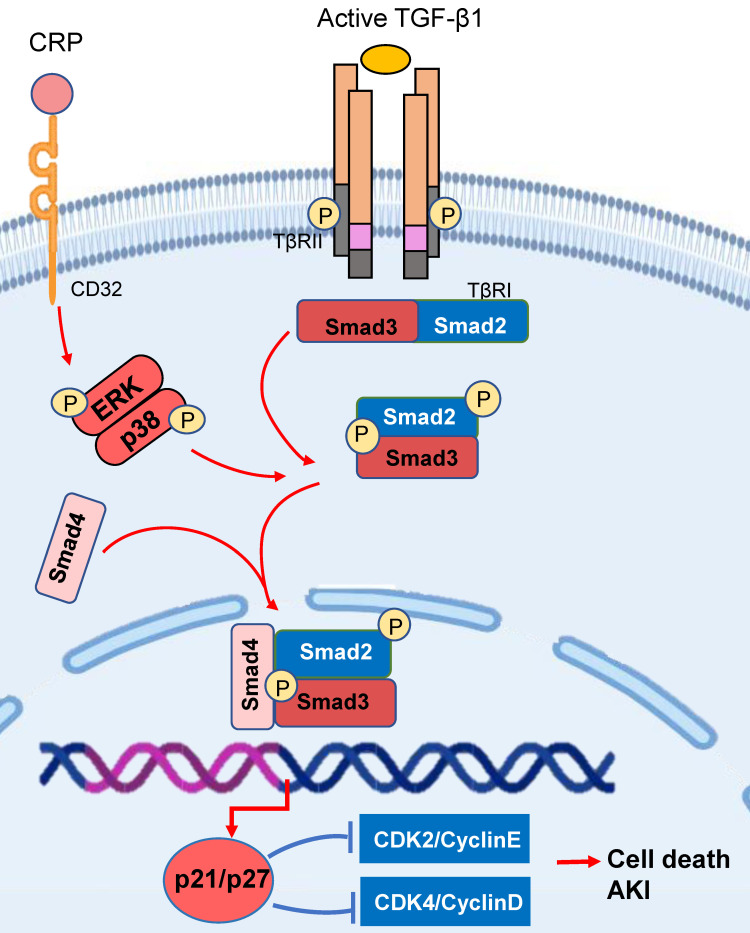
** Smad3 triggers the cell death pathways in acute kidney injury.** Smad3 can bind and activate the p21/p27 to cause tubular epithelial cell death via the G1 cell-cycle arrest mechanism in response to TGF-β1 and CRP under various kidney disease conditions. Red arrows/symbols represent pathogenic or positive regulation pathway, while blue lines/symbols indicate protective or negative regulation pathways.

Increasing evidence shows that AKI is a major cause of CKD. Smad3 also plays a driving role in AKI by triggering the cell death pathways (Figure 3). Recent studies identified that Smad3 can bind and induce expression of cyclin-dependent kinase inhibitors (CDKIs) including p21/p27 to cause tubular epithelial cell death via the G1 cell-cycle arrest mechanism 68, 69. It has been reported that CRP can induce the early activation of Smad3 signaling via the CD32-ERK1/2 and p38-dependent mechanism 41. Thus, mice overexpressing the human CRP develop more severe AKI by activating Smad3-dependent cell death pathway 68, 70, which is reversed by targeting this pathway with a pharmacological Smad3 inhibitor 68. In addition, activation of TGF-β/Smad3 signaling also plays a key role in the cell senescence during the development of aging kidney, which is mediated via the p16/p21-dependent mechanism 71. In podocyte-specific TGF-β overexpressing mice, over-activation of TGF-β/Smad3 signaling is involved in the cell senescence via p16 translocation and p21 induction 72. All these studies suggest that activation of TGF-β/Smad3-p16/p21pathway may not only cause cell death in AKI and but also involves in renal aging and fibrosis in CKD.

Necroptosis is another cell death pathway leading to AKI 73. Necroptotic cells can release the components such as high mobility group protein to induce severe necroinflammation 74. Emerging evidence shows that RIPK1, RIPK3, and MLKL are central regulators in the necroptotic pathway 75, 76. It has been shown that Smad3 can interact with RIPK and thus loss of Smad3 significantly blocks RIPK-mediated programmed cell death and inflammation 77.

AKI is also a common clinical feature in critically ill patients with COVID-19, particularly in those with inflammatory stress and underlying disease conditions 78. Strikingly, our most recent study discovered that among SARS-CoV-2 proteins, SARS-CoV-2 N protein is pathogenic for AKI as kidney-specifically overexpressing SARS-CoV-2 N protein can directly induce AKI and promote severe AKI under ischemic conditions 79. Mechanically, we uncover that SARS-CoV-2 N protein can bind and activate Smad3 signaling to trigger the p21-dependent cell death pathway via the G1 cell cycle arrest mechanism 79, 80. Thus, targeting Smad3 by genic deletion of Smad3 or pharmacological inhibition of Smad3 signaling can protect against SARS-CoV-2 N-induced AKI 79. As Smad3 is a key mediator of renal fibrosis 51, it is highly possible that after SARS-CoV-2 infection, intracellular release of SARS-CoV-2 N protein can bind and activate TGF-β/Smad3 signaling to induce the cell death pathway to trigger renal inflammation and “cytokine storm”, resulting in AKI. It is also possible that activation of renal TGF-β/Smad3 signaling results in COVID-19 associated fibrosis including lung and renal fibrosis 81. Thus, targeting Smad3 may represent as a novel and promising therapeutic strategy for critically ill COVID-19 patients.

## Regulation of Smad3-dependent microRNAs and lncRNA in renal inflammation and fibrosis

Noncoding RNAs, including miRNAs, siRNAs, piwi-interacting RNAs, and various types of lncRNAs, are involved in the progression of kidney diseases. MiRNAs are small noncoding RNAs of approximately 22 nucleotides in length, and they bind to the 30-untranslated region of target genes to regulate gene expression by translational repression or induction of mRNA degradation. MiRNAs are important regulators of cell proliferation, differentiation, and apoptosis 82, 83.

**Figure 4:**
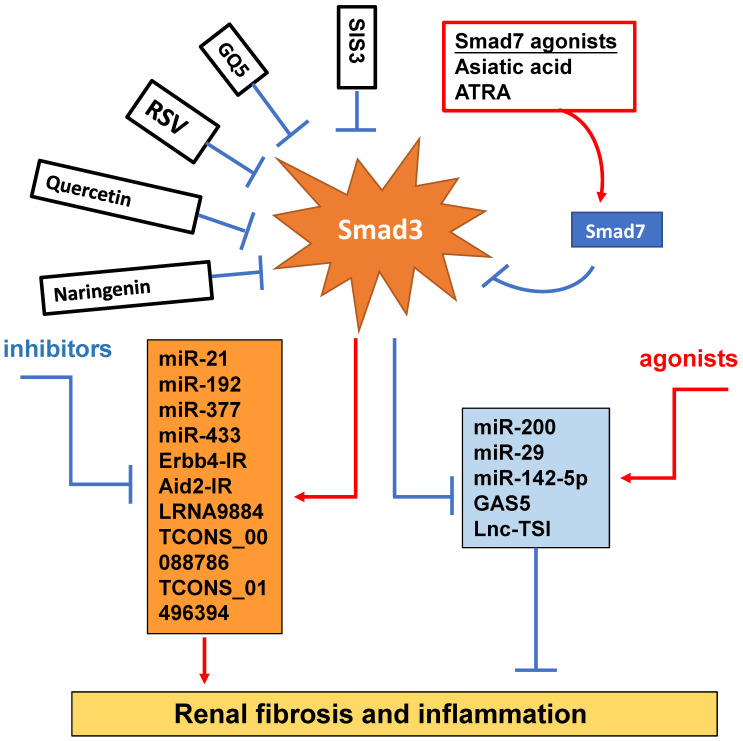
**Smad3-dependent miRNAs and lncRNAs related to renal fibrosis and inflammation and Smad3 targeting therapy for renal fibrosis and inflammation.** Smad3 positively regulate pro-fibrogenic or pro-inflammatory miRNAs and lncRNAs, but negatively regulate those to mediate renal fibrosis and inflammation. Specifically targeting Smad3 directly with inhibitors or Smad7 agonists or indirectly to its downstream non-coding RNAs may be the potential therapeutic strategies for renal fibrosis and inflammation. Red arrows/symbols represent pathogenic or positive regulation pathway, while blue lines/symbols indicate protective or negative regulation pathways.

It has been well documented that TGF-β/Smad signaling plays a critical regulatory role in renal inflammation and fibrosis by regulating a number of miRNAs 6, 84 (Figure 4). TGF-β1/Smad3 signaling is capable of inducing miR-21 85, miR-192 86 and miR-377 87, but it reduces the expression of miR-200 88 and miR-29 families 89. MiR-21 is reported to play a role in the inflammatory response, immunomodulation and fibrotic disorders 83. MiR-21 is upregulated in animal models with progressive renal fibrosis and inhibition of miR-21 ameliorates fibrosis in obstructive and diabetic kidney diseases 90-92. Mechanistically, Smad3 can directly interact with miR-21 and induce the expression of miR-21 in response to TGF-β1 and AGEs 85, 93. MiR-192 is also a downstream mediator of TGF-β/Smad3 in renal fibrosis. Smad3 could physically interact with the promoter region of miR-192 to induce its expression 94. Thus, TGF-β-induced tubular miR-192 expression is Smad3-dependent because knockdown of Smad3 can block TGF-β1-induced tubular miR-192 expression and renal fibrosis 94. This observation is further confirmed in Smad3 KO MEF cells and in the UUO kidney in which deletion of Smad3 inhibits renal miR-192 and progressive renal fibrosis 94. MiR-433 also acts as a downstream mediator of TGF-β/Smad3-driven renal fibrosis by targeting the antizyme inhibitor Azin1 as silencing miR-433 upregulates Azin1 and inhibits renal fibrosis in a mouse model of UUO 95. In contrast, miR-29 is protective in renal fibrosis and is negatively regulated by TGF-β via Smad3 as the promoter region of miR-29 contains at least two conserved Smad3 binding-sites and Smad3 could physically interact with the promoter region of miR-29 89.Thus, Smad3 acts as a suppressor to negatively regulate miR-29 expression during TGF-β-mediated fibrosis, and deletion of Smad3 enhances miR-29b expression, thereby inhibiting collagen matrix expression under high TGF-β1 and diabetic conditions 89, 96, 97.

Increasing evidence shows that renal inflammation and fibrosis are also tightly regulated by a few Smad3-dependent long noncoding RNAs (lncRNAs) 98 (Figure 4). Indeed, research into the lncRNAs is more promising for a better understanding of the pathogenic mechanisms of kidney diseases. Compared to miRNAs, lncRNAs are transcripts with lengths exceeding 200 nucleotides without protein-coding functions. LncRNA regulates both target DNAs/RNAs and proteins transcriptionally or post-transcriptionally 99. By using the high-throughput RNA sequencing, we identify that 413 lncRNAs (plus or minus two-fold to ninefold) are differentially expressed in WT and Smad3 KO kidneys of anti-glomerular basement membranous glomerulonephritis (anti-GBM GN), of them, 21 Smad3-dependent common lncRNAs are altered in both UUO and anti-GBM GN models 100. Erbb4-IR is a novel Smad3-dependent lncRNA and is highly upregulated in the UUO and diabetic kidneys with progressive renal fibrosis 101, 102. The functional role of Erbb4-IR in renal fibrosis is demonstrated by silencing this lncRNA to protect kidneys from both UUO and diabetic injury 101, 102. Mechanistically, Erbb4-IR mediates renal fibrosis in the UUO kidney and diabetic nephropathy by targeting renal Smad7 and miR-29b 101, 102. The Arid2-IR is also another novel Smad3-related lncRNA. Arid2-IR is one of the most highly upregulated lncRNAs in the UUO kidney with progressive renal inflammation and fibrosis. The promoter region of Arid2-IR contains a Smad3 binding site and thus deletion of Smad3 gene completely blocked upregulation of Arid2-IR in the UUO kidney, suggesting a positive regulatory role for Smad3 in Arid2-IR expression during renal inflammation. Further study reveals that Arid2-IR mediates renal inflammation via the NF-κB-dependent mechanism and thus Arid2-IR may be a downstream mediator of Smad3 and functions to promote NF-κB-driven renal inflammation without effect on TGF-β/Smad3-mediated renal fibrosis in a mouse model of obstructive nephropathy and *in vitro* 103. LRNA9884 has been shown to play a proinflammatory role and mediates renal inflammation in db/db mice via a MCP-1-dependent mechanism 104. Further study also shows that LRNA9884 can induce renal inflammation in AKI mouse model by upregulating macrophage migration inhibitory factor via the NF-κB-dependent mechanism 104, 105. Lnc-TSI is a lncRNA which is specifically expressed in the fibrotic kidney and negatively correlated with the severity of renal fibrosis. Interestingly, repeated renal biopsy reveals that the lower expression levels of renal Lnc-TSI at the initial kidney biopsy is associated with a more pronounced decline in renal function and fibrosis 4 years later, suggesting that kidney-enriched Lnc-TSI may protect against renal fibrogenesis 106. It is possible that Lnc-TSI may function as a negative regulator of TGF-β1/Smad3 signaling as overexpression of Lnc-TSI can block Smad3 activation and interaction with TβRI by binding with the MH2 domain of Smad3 106. Thus, Smad3 suppresses Lnc-TSI expression through binding with the promoter of Lnc-TSI under TGF-β1 stimulation 106. GAS5 is also an anti-fibrotic lncRNA and is highly expressed in normal renal tubular epithelial cells but lost in the fibrotic kidney at 7 days after UUO surgery 107. GAS5 is tightly regulated by Smad3 and thus deletion of Smad3 dramatically inhibits GAS5 in the kidneys of UUO mice *in vivo* and in TGF-β-stimulated MEFs *in vitro* 107. In addition, TCONS_00088786 and TCONS_01496394 are TGF-β/Smad3-associated lncRNAs as they contain potential binding sites for Smad3 and silencing TCONS_00088786 inhibits renal interstitial fibrosis in UUO rat model 108.

## Therapeutic potential for renal inflammation and fibrosis by targeting Smad3 signaling

Although TGF-β/Smad3 has been considered as a major pathway for fibrogenesis, the diverse roles of this pathway in renal inflammation and fibrosis have hampered the development of anti-TGF-β treatment in general 4-7. The failure of anti-TGF-β antibodies-based therapy in recent clinical trials has proved that treatment by targeting upstream TGF-β signaling may not be a good strategy for the treatment of kidney diseases 109, 110. Disappointingly, treatment with a humanized monoclonal neutralizing antibody against TGF-β1 (LY2382770) for patients with diabetic nephropathy shows no efficacy on the improvements of renal dysfunction including serum creatinine, estimated GFR (eGFR), and proteinuria 109. Similarly, the use of Fresolimumab (another humanized monoclonal antibody) that inhibits all three isoforms of TGF-β also fails to achieve the endpoints of proteinuria reduction in patients with FSGS 110. It is highly possible that blockade of the entire TGF-β1 signaling may also promote inflammation as TGF-β1 is a potent anti-inflammatory cytokine 4, 111-113. Thus, targeting the downstream TGF-β signaling molecules specifically related to fibrosis or inflammation could be a better therapeutic approach. Many studies have reported that Smad3 can directly bind to the DNA sequences to regulate expression of several fibrogenic genes and the process of EMT and MMT 51, 84, 114. Thus, treatment should aim to specifically target Smad3, and its dependent genes directly related to fibrogenesis or inflammation, rather than the entire TGF-β signaling (Figure 4). SIS3 is a small molecule capable of directly suppressing Smad3-mediated expression of collagens matrix 115 and inhibiting the accumulation of α-SMA^+^ myofibroblasts in the fibrotic kidney by blocking Smad3-dependent myofibroblasts transdifferentiation including MMT 28, 31, 32. Treatment with SIS3 may also block Smad3-dependent auto-induction of TGF-β1 via a positive feedback loop of TGF-β1/Smad3 signaling 116. Furthermore, SIS3 can ameliorate renal inflammation and tubular apoptosis in both AKI and UUO kidneys 116, 117. Excitingly, our recent study also discovered the therapeutic effect of SIS3 on SARS-CoV-2 N-induced AKI by inhibiting Smad3-dependent p21-mediated cell death pathway 79. Thus, specifically targeting Smad3 may be a novel therapeutic approach for kidney diseases.

In the fibrotic and inflammatory kidney, overactive Smad3 signaling is associated with the loss of renal Smad7 20, 21, 114. Thus rebalancing Smad3/Smad7 signaling by either inhibiting Smad3 and/or activating Smad7 may be a better approach for the development of effective and specific therapy for kidney diseases 7. This is supported by many studies in which overexpressing renal Smad7 can block TGF-β/Smad3-mediated renal fibrosis and NF-κB-driven renal inflammation in diabetic kidney disease 22, 118, crescentic glomerulonephritis 119, UUO 120, AKI 69, and hypertensive nephropathy 22, 120, 121. Recently, we also identified that naringenin (NG), a flavonoid from grapefruit and citrus fruits 122, functions as a Smad3 inhibitor, whereas asiatic acid (AA), a purified compound from Centella asiatica 123, is a Smad7 agonist. The combination of these two purified traditional Chinese medicine compounds significantly rebalances the Smad3/Smad7 signaling and thus additively enhances the inhibitory effect on TGF-β1/Smad3 signaling and renal fibrosis *in vitro* and *in vivo* 124. Quercetin is also functioning to inhibit Smad3 signaling and has been shown to have therapeutic effect on cisplatin-induced AKI 125, 126. GQ5 (a small compound isolated from Resina Toxicodendron) can block the interaction of Smad3 with TβRI and attenuates renal fibrosis 127. Resveratrol (RSV) is a natural plant polyphenol with anti-fibrotic and anti-inflammatory properties. RSV treatment can significantly activate Sirt1 to suppress Smad3 acetylation and the TGF-β1-induced fibrotic response in the remnant kidney of 5/6 nephrectomized rodents, obstructed kidney model or in cultured cells following TGF-β1 treatment 128. All-trans retinoic acid (ATRA), an active metabolite of vitamin A, belongs to the retinoids family. Treatment with ATRA inactivates Smad3 signaling and protects against the diabetic kidney disease by upregulating renal Smad7 129.

Increasing evidence also shows that specifically altering the Smad3-dependent microRNAs or lncRNAs related to fibrogenesis or inflammation locally in the diseased kidney could be a better therapeutic approach for combating kidney disorders. As described elsewhere 84, 98 and illustrated in Figure 4, epigenetically targeting miR-21 85, 91, miR-192 86, 94, miR-433 95, miR-29 96, 97 and miR-200 family 88, Erbb4-IR 102, LRNA9884 104, 105, Arid2-IR 103, and Lnc-TSI 106 have been shown to be a novel and specific anti-fibrosis and anti-inflammation therapy for kidney diseases. More excitingly, we have developed a kidney-specifically genes delivery system by using non-invasive ultrasound-microbubble-technique, which can effectively transfer the genes or miRNAs/lncRNAs into the kidney to block renal inflammation and fibrosis without detectable side effects 85, 95, 96, 102, 103.

## Conclusions

The current advances in research into the regulation of TGF-β signaling and particularly the Smad3-dependent noncoding RNAs have improved our understanding of the molecular mechanisms of renal inflammation and fibrosis in kidney diseases. In term of renal fibrosis, Smad3 is pathogenic and overreactive, whereas Smad7 is protective but lost in the fibrotic kidney. Thus, rebalancing Smad3/Smad7 signaling may be a better therapeutic approach for combating kidney diseases. In addition, epigenetic identification of Smad3-dependent non-coding RANs that specifically regulate renal inflammation and fibrosis may be the key step forwards the development of effective therapy for kidney diseases. It is also highly possible that targeting Smad3 may be a novel therapeutic potential for AKI by protecting kidney cell death from G1 cell cycle arrest.
